# Supplementary material for: Cell types and neuronal circuitry underlying female aggression in Drosophila
Source: eLife. 2020 Nov 3;9:e58942. doi: 10.7554/eLife.58942 (PMC7787668; doi:10.7554/eLife.58942)
Supplement: Supplementary file 3. [file elife-58942-supp3.docx]

**Sample Size and Statistics**

**Figure 1G – H**

Analysis – (G) Total time an individual fly spent performing aggressive behaviors during each of four 30-second periods: prior to, during, immediately following, and 30-60 sec after the stimulus. (H) Total time an individual spent performing aggressive behaviors over a 30-minute trial. Biological repeats for Figure 1H are shown in Figure 1.11.

All are biological replicates and data are representative of at least two independent trials per experiment.

| Figure | Condition | # | Sample Size | Statistical  Test | Comparison | P-value |
| --- | --- | --- | --- | --- | --- | --- |
| 1G | 20xChrimson | 1 | 71 | Kruskal- | During: |  |
|  | aIPgSS1  EmptySS>Chrimson | 2  3 | 65  78 | Wallis  (P < 0.0001) | 1 v 4  2 v 4 | <0.0001  <0.0001 |
|  | aIPgSS1>Chrimson  20xChrimson | 4  5 | 100  71 | Kruskal- | 3 v 4  0 – 30s: | <0.0001 |
|  | aIPgSS1  EmptySS>Chrimson  aIPgSS1>Chrimson  20xChrimson  aIPgSS1  EmptySS>Chrimson  aIPgSS1>Chrimson | 6  7  8  9  10  11  12 | 65  78  100  71  65  78  100 | Wallis  (P < 0.0001)  Kruskal-  Wallis  (P < 0.0001) | 6 v 5  7 v 5  8 v 5  30 – 60s:  10 v 9  11 v 9  12 v 9 | <0.0001  0.0005  <0.0001  <0.0001  0.0013  0.0278 |
|  |  |  |  |  |  |  |

| Figure | Condition | # | Sample Size | Statistical  Test | Comparison | P-value |
| --- | --- | --- | --- | --- | --- | --- |
| 1H | aIPgSS1>GFP | 1 | 54 | Kruskal-Wallis | 1 v 2 | 0.1436 |
|  | EmptySS>TNTe  aIPgSS1>TNTe | 2  3 | 28  30 | (P < 0.0001) | 1 v 3  2 v 3 | <0.0001  0.0048 |
|  |  |  |  |  |  |  |

**Figure 1-supplement 6**

Analysis – (B, D, H) The percentage of flies in each experiment engaging in touching (B), chasing (D), and aggressive (H) behaviors. (F) Velocity during the stimulus period and in the 30-60 seconds following the stimulus. (G) Difference in the average number of flies within two body lengths over the 30-seconds prior to and during the stimulus.

All are biological replicates and data are representative of at least two independent trials per experiment. Sample size for B, D, F, and H are displayed per experiment and each experiment consists of approximately 15 flies.

| Figure | Condition | # | Sample Size | Statistical  Test | Comparison | P-value |
| --- | --- | --- | --- | --- | --- | --- |
| 1.6B | EmptySS>Chrimson | 1 | 5 | Mann-Whitney | During: |  |
|  | aIPgSS1>Chrimson | 2 | 7 |  | 1 v 2 | 0.0025 |
|  |  |  |  |  |  |  |

| Figure | Condition | # | Sample Size | Statistical  Test | Comparison | P-value |
| --- | --- | --- | --- | --- | --- | --- |
| 1.6D | EmptySS>Chrimson | 1 | 5 | Mann-Whitney | During: |  |
|  | aIPgSS1>Chrimson | 2 | 7 |  | 1 v 2 | 0.0025 |
|  |  |  |  |  |  |  |

| Figure | Condition | # | Sample Size | Statistical  Test | Comparison | P-value |
| --- | --- | --- | --- | --- | --- | --- |
| 1.6F | EmptySS>Chrimson | 1 | 5 | Mann-Whitney | During: |  |
|  | aIPgSS1>Chrimson | 2 | 7 |  | 1 v 2 | 0.0025 |
|  |  |  |  |  |  |  |

| Figure | Condition | # | Sample Size | Statistical  Test | Comparison | P-value |
| --- | --- | --- | --- | --- | --- | --- |
| 1.6G | EmptySS>Chrimson | 1 | 78 | Mann-Whitney | During: |  |
|  | aIPgSS1>Chrimson | 2 | 100 |  | 1 v 2 | 0.0146 |
|  |  |  |  |  |  |  |

| Figure | Condition | # | Sample Size | Statistical  Test | Comparison | P-value |
| --- | --- | --- | --- | --- | --- | --- |
| 1.6H | EmptySS>Chrimson | 1 | 5 | Mann-Whitney | During: |  |
|  | aIPgSS1>Chrimson | 2 | 7 |  | 1 v 2 | 0.0025 |
|  |  |  |  |  |  |  |

**Figure 1-supplement 7**

Analysis – (A – B) The percentage of flies in each experiment engaging in aggressive behaviors.

All are biological replicates and data are representative of at least two independent trials per experiment. Sample size is displayed per experiment and each experiment consists of approximately 15 flies.

| Figure | Condition | # | Sample Size | Statistical  Test | Comparison | P-value |
| --- | --- | --- | --- | --- | --- | --- |
| 1.7B | EmptySS>Chrimson - | 1 | 7 | Kruskal-Wallis | During: |  |
|  | aIPgSS1>Chrimson -  EmptySS>Chrimson + | 2  3 | 7  7 | (P = 0.0001) | 1 v 2  3 v 4 | 0.5772  0.0006 |
|  | aIPgSS1>Chrimson + | 4 | 8 |  |  |  |
|  |  |  |  |  |  |  |

**Figure 1-supplement 9**

Analysis – (C) Average percentage of flies performing aggressive behaviors over the 30-second period during stimulus delivery. (D) Average percentage of flies performing aggressive behaviors over the 30-second period before 1 Hz, the 60-second periods between subsequent stimuli, and the 30-seconds after 50 Hz stimulation.

All are biological replicates and data are representative of at least two independent trials per experiment. Sample size is displayed per experiment and each experiment consists of approximately 15 flies.

| Figure | Condition | # | Sample Size | Statistical  Test | Comparison | P-value |
| --- | --- | --- | --- | --- | --- | --- |
| 1.10C | Empty>Chrimson 5Hz | 1 | 4 | Mann-Whitney | 1 v 2 | 0.0095 |
|  | aIPgSS1>Chrimson 5Hz  EmptySS>Chrimson 10Hz | 2  3 | 6  4 | Mann-Whitney | 3 v 4 | 0.0095 |
|  | aIPgSS1>Chrimson 10Hz  EmptySS>Chrimson 20Hz  aIPgSS1>Chrimson 20Hz  Empty>Chrimson 30Hz  aIPgSS1>Chrimson 30Hz  Empty>Chrimson 50Hz  aIPgSS1>Chrimson 50Hz | 4  5  6  7  8  9  10 | 6  4  6  4  6  4  6 | Mann-Whitney  Mann-Whitney  Mann-Whitney | 5 v 6  7 v 8  9 v 10 | 0.0095  0.0095  0.0095 |
|  |  |  |  |  |  |  |

| Figure | Condition | # | Sample Size | Statistical  Test | Comparison | P-value |
| --- | --- | --- | --- | --- | --- | --- |
| 1.10D | Empty>Chrimson 10-Hz | 1 | 4 | Mann-Whitney | 1 v 2 | 0.0190 |
|  | aIPgSS1>Chrimson 10-Hz  EmptySS>Chrimson 20-Hz | 2  3 | 6  4 | Mann-Whitney | 3 v 4 | 0.3524 |
|  | aIPgSS1>Chrimson 20-Hz  EmptySS>Chrimson 30-Hz  aIPgSS1>Chrimson 30-Hz  Empty>Chrimson 50-Hz  aIPgSS1>Chrimson 50-Hz | 4  5  6  7  8 | 6  4  6  4  6 | Mann-Whitney  Mann-Whitney | 5 v 6  7 v 8 | 0.0095  0.0095 |
|  |  |  |  |  |  |  |

**Figure 1 – figure supplement 10**

Analysis – (A – C) Total time an individual spent performing aggressive behaviors over a 30-minute trial.

All are biological replicates and data are representative of at least two independent trials per experiment.

| Figure | Condition | # | Sample Size | Statistical  Test | Comparison | P-value |
| --- | --- | --- | --- | --- | --- | --- |
| 1.10A | EmptySS>TNTe | 1 | 70 | Mann-Whitney | 1 v 2 | <0.0001 |
|  | aIPgSS4>TNTe | 2 | 102 |  |  |  |
|  |  |  |  |  |  |  |

| Figure | Condition | # | Sample Size | Statistical  Test | Comparison | P-value |
| --- | --- | --- | --- | --- | --- | --- |
| 1.10B | EmptySS>TNTe | 1 | 7 | Mann-Whitney | 1 v 2 | 0.0047 |
|  | aIPgSS4>TNTe | 2 | 6 |  |  |  |
|  |  |  |  |  |  |  |

| Figure | Condition | # | Sample Size | Statistical  Test | Comparison | P-value |
| --- | --- | --- | --- | --- | --- | --- |
| 1.10C | EmptySS>TNTe | 1 | 84 | Mann-Whitney | 1 v 2 | 0.0003 |
|  | aIPgSS5>TNTe | 2 | 36 |  |  |  |
|  |  |  |  |  |  |  |

**Figure 1 – figure supplement 11**

Analysis – (A – B) Total time an individual spent performing aggressive behaviors over a 30-minute trial. (C – D) Average velocity over a 30-minute trial.

A – B are the biological replicates for Figure 1H.

| Figure | Condition | # | Sample Size | Statistical  Test | Comparison | P-value |
| --- | --- | --- | --- | --- | --- | --- |
| 1.11A | aIPgSS1>GFP | 1 | 52 | Kruskal-Wallis | 1 v 3 | 0.0253 |
|  | EmptySS>TNTe  aIPgSS1>TNTe | 2  3 | 84  62 | (P = 0.0058) | 2 v 3 | 0.0103 |
|  |  |  |  |  |  |  |

| Figure | Condition | # | Sample Size | Statistical  Test | Comparison | P-value |
| --- | --- | --- | --- | --- | --- | --- |
| 1.11B | aIPgSS1>ImpTNTe | 1 | 110 | Kruskal-Wallis | 1 v 2 | 0.0017 |
|  | EmptySS>TNTe  aIPgSS1>TNTe | 2  3 | 56  74 | (P < 0.0001) | 1 v 3  2 v 3 | <0.0001  0.0099 |
|  |  |  |  |  |  |  |

| Figure | Condition | # | Sample Size | Statistical  Test | Comparison | P-value |
| --- | --- | --- | --- | --- | --- | --- |
| 1.11C | aIPgSS1>GFP | 1 | 54 | Kruskal-Wallis | 1 v 2 | 0.2899 |
|  | EmptySS>TNTe  aIPgSS1>TNTe | 2  3 | 28  30 | (P = 0.0034) | 1 v 3  2 v 3 | 0.0797  0.0025 |
|  |  |  |  |  |  |  |

| Figure | Condition | # | Sample Size | Statistical  Test | Comparison | P-value |
| --- | --- | --- | --- | --- | --- | --- |
| 1.11D | aIPgSS1>GFP | 1 | 52 | Kruskal-Wallis | 1 v 2 | 0.0312 |
|  | EmptySS>TNTe  aIPgSS1>TNTe | 2  3 | 84  62 | (P = 0.0220) | 1 v 3  2 v 3 | >0.9999  0.1402 |
|  |  |  |  |  |  |  |

**Figure 2**

Analysis – (A – C) Total time an individual spent performing aggressive behaviors in a 16 mm arena over the 30 second period prior to or during stimulation. (A' – C') Amount of time during a 30 second 0.1 mW/mm^2^ continuous stimulation period until first aggressive encounter.

All are biological replicates and data are representative of at least two independent trials per experiment. For p-values of representative repeat, see Figure 2 figure legend.

| Figure | Condition | # | Sample Size | Statistical  Test | Comparison | P-value |
| --- | --- | --- | --- | --- | --- | --- |
| 2A | EmptySS>Chrimson | 1 | 22 | Mann-Whitney | During: |  |
|  | aIPgSS1>Chrimson | 2 | 14 |  | 1 v 2 | <0.0001 |
|  |  |  |  |  |  |  |

| Figure | Condition | # | Sample Size | Statistical  Test | Comparison | P-value |
| --- | --- | --- | --- | --- | --- | --- |
| 2B | EmptySS>Chrimson | 1 | 8 | Mann-Whitney | During: |  |
|  | aIPgSS1>Chrimson | 2 | 7 |  | 1 v 2 | 0.0014 |
|  |  |  |  |  |  |  |

| Figure | Condition | # | Sample Size | Statistical  Test | Comparison | P-value |
| --- | --- | --- | --- | --- | --- | --- |
| 2C | EmptySS>Chrimson | 1 | 7 | Mann-Whitney | During |  |
|  | aIPgSS1>Chrimson | 2 | 7 |  | 1 v 2 | 0.0256 |
|  |  |  |  |  |  |  |

| Figure | Condition | # | Sample Size | Statistical  Test | Comparison | P-value |
| --- | --- | --- | --- | --- | --- | --- |
| 2A' | EmptySS>Chrimson | 1 | 22 | Mann-Whitney | 1 v 2 | <0.0001 |
|  | aIPgSS1>Chrimson | 2 | 14 |  |  |  |
|  |  |  |  |  |  |  |

| Figure | Condition | # | Sample Size | Statistical  Test | Comparison | P-value |
| --- | --- | --- | --- | --- | --- | --- |
| 2B' | EmptySS>Chrimson | 1 | 8 | Mann-Whitney | 1 v 2 | 0.0014 |
|  | aIPgSS1>Chrimson | 2 | 7 |  |  |  |
|  |  |  |  |  |  |  |

| Figure | Condition | # | Sample Size | Statistical  Test | Comparison | P-value |
| --- | --- | --- | --- | --- | --- | --- |
| 2C' | EmptySS>Chrimson | 1 | 7 | Mann-Whitney | 1 v 2 | 0.0693 |
|  | aIPgSS1>Chrimson | 2 | 7 |  |  |  |
|  |  |  |  |  |  |  |

**Figure 2 – figure supplement 1**

Analysis – (A – C) Total time an individual spent performing aggressive behaviors in a 16 mm arena over the 30 second period prior to or during stimulation. (A' – C') Amount of time during a 30 second 0.1 mW/mm^2^ continuous stimulation period until first aggressive encounter.

All are biological replicates and data are representative of at least two independent trials per experiment.

| Figure | Condition | # | Sample Size | Statistical  Test | Comparison | P-value |
| --- | --- | --- | --- | --- | --- | --- |
| 2.1A | EmptySS>Chrimson | 1 | 8 | Mann-Whitney | During: |  |
|  | aIPgSS3>Chrimson | 2 | 20 |  | 1 v 2 | <0.0001 |
|  |  |  |  |  |  |  |

| Figure | Condition | # | Sample Size | Statistical  Test | Comparison | P-value |
| --- | --- | --- | --- | --- | --- | --- |
| 2.1B | EmptySS>Chrimson | 1 | 7 | Mann-Whitney | During: |  |
|  | aIPgSS3>Chrimson | 2 | 10 |  | 1 v 2 | 0.0414 |
|  |  |  |  |  |  |  |

| Figure | Condition | # | Sample Size | Statistical  Test | Comparison | P-value |
| --- | --- | --- | --- | --- | --- | --- |
| 2.1C | EmptySS>Chrimson | 1 | 8 | Mann-Whitney | During |  |
|  | aIPgSS3>Chrimson | 2 | 10 |  | 1 v 2 | 0.0007 |
|  |  |  |  |  |  |  |

| Figure | Condition | # | Sample Size | Statistical  Test | Comparison | P-value |
| --- | --- | --- | --- | --- | --- | --- |
| 2.1A' | EmptySS>Chrimson | 1 | 8 | Mann-Whitney | 1 v 2 | <0.0001 |
|  | aIPgSS3>Chrimson | 2 | 20 |  |  |  |
|  |  |  |  |  |  |  |

| Figure | Condition | # | Sample Size | Statistical  Test | Comparison | P-value |
| --- | --- | --- | --- | --- | --- | --- |
| 2.1B' | EmptySS>Chrimson | 1 | 7 | Mann-Whitney | 1 v 2 | 0.0288 |
|  | aIPgSS3>Chrimson | 2 | 10 |  |  |  |
|  |  |  |  |  |  |  |

| Figure | Condition | # | Sample Size | Statistical  Test | Comparison | P-value |
| --- | --- | --- | --- | --- | --- | --- |
| 2.1C' | EmptySS>Chrimson | 1 | 8 | Mann-Whitney | 1 v 2 | 0.0003 |
|  | aIPgSS3>Chrimson | 2 | 10 |  |  |  |
|  |  |  |  |  |  |  |

**Figure 2 – figure supplement 2**

Analysis – Average velocity over the 30-seconds either prior to or during the stimulus.

All are biological replicates and data are representative of at least two independent trials per experiment.

| Figure | Condition | # | Sample Size | Statistical  Test | Comparison | P-value |
| --- | --- | --- | --- | --- | --- | --- |
| 2.2 | aIPgSS1>ChrimsonPreStim | 1 | 11 | Mann-Whitney | 1 v 2 | 0.7969 |
|  | aIPgSS1>ChrimsonStim | 2 | 11 |  |  |  |
|  |  |  |  |  |  |  |

**Figure 2 – figure supplement 3**

Analysis – Amount of time until first copulation event following a continuous stimulus.

All are biological replicates and data are representative of at least two independent trials per experiment.

| Figure | Condition | # | Sample Size | Statistical  Test | Comparison | P-value |
| --- | --- | --- | --- | --- | --- | --- |
| 2.3 | EmptySS>Chrimson | 1 | 16 | Mann-Whitney | 1 v 2 | 0.7795 |
|  | aIPgSS1>Chrimson | 2 | 18 |  |  |  |
|  |  |  |  |  |  |  |

**Figure 4**

Analysis – (C) Total time an individual spent performing aggressive behaviors during each of four 30-second periods: prior to, during, immediately following, and 30-60 sec after the stimulus.

All are biological replicates and data are representative of at least three independent trials per experiment.

| Figure | Condition | # | Sample Size | Statistical  Test | Comparison | P-value |
| --- | --- | --- | --- | --- | --- | --- |
| 4C | 20xChrimson | 1 | 29 | Kruskal- | During: |  |
|  | pC1dSS1  EmptySS>Chrimson | 2  3 | 48  46 | Wallis  (P < 0.0001) | 1 v 4  2 v 4 | 0.0017  <0.0001 |
|  | pC1dSS1>Chrimson  20xChrimson | 4  5 | 53  29 | Kruskal- | 3 v 4  0 – 30s: | <0.0001 |
|  | pC1dSS1  EmptySS>Chrimson  pC1dSS1>Chrimson  20xChrimson  pC1dSS1  EmptySS>Chrimson  pC1dSS1>Chrimson | 6  7  8  9  10  11  12 | 48  46  53  29  48  46  53 | Wallis  (P = 0.0242)  Kruskal-  Wallis  (P = 0.0036) | 6 v 5  7 v 5  8 v 5  30 – 60s:  10 v 9  11 v 9  12 v 9 | 0.8066  0.0259  >0.9999  >0.9999  0.2092  0.7593 |
|  |  |  |  |  |  |  |

**Figure 4 – figure supplement 5**

Analysis – (A) Average percentage of flies engaging in aggressive behaviors over the 30-second period prior to or during stimulus delivery. (C', D') Average percentage of flies touching (C') or chasing (D') over the 30-second period prior to or during the stimulus delivery.

All are biological replicates and data are representative of at least three independent trials per experiment. Sample size is displayed per experiment and each experiment consists of approximately 15 flies.

| Figure | Condition | # | Sample Size | Statistical  Test | Comparison | P-value |
| --- | --- | --- | --- | --- | --- | --- |
| 4.5A | EmptySS>Chrimson | 1 | 5 | Mann-Whitney | During: |  |
|  | pC1dSS1>Chrimson | 2 | 5 |  | 1 v 2 | 0.0079 |
|  |  |  |  |  |  |  |

| Figure | Condition | # | Sample Size | Statistical  Test | Comparison | P-value |
| --- | --- | --- | --- | --- | --- | --- |
| 4.5C' | EmptySS>Chrimson | 1 | 5 | Mann-Whitney | During: |  |
|  | pC1dSS1>Chrimson | 2 | 5 |  | 1 v 2 | 0.0079 |
|  |  |  |  |  |  |  |

| Figure | Condition | # | Sample Size | Statistical  Test | Comparison | P-value |
| --- | --- | --- | --- | --- | --- | --- |
| 4.5D' | EmptySS>Chrimson | 1 | 5 | Mann-Whitney | During: |  |
|  | pC1dSS1>Chrimson | 2 | 5 |  | 1 v 2 | 0.0159 |
|  |  |  |  |  |  |  |

**Figure 4 – figure supplement 6**

Analysis – (B) Average percentage of flies engaging in aggressive behaviors over the 30 second period prior to or during the delivery of a stimulus.

All are biological replicates and data are representative of at least two independent trials per experiment. Sample size is displayed per experiment and each experiment consists of approximately 15 flies.

| Figure | Condition | # | Sample Size | Statistical  Test | Comparison | P-value |
| --- | --- | --- | --- | --- | --- | --- |
| 4.6B | EmptySS>Chrimson | 1 | 1 | Kruskal-Wallis | During: |  |
|  | pC1dSS1>Chrimson  pC1eSS1>Chrimson | 2  3 | 3  3 | (P = 0.0146) | 2 v 4 | >0.9999 |
|  | pC1d/eSS>Chrimson | 4 | 5 |  |  |  |
|  |  |  |  |  |  |  |
|  |  |  |  |  |  |  |

**Figure 4 – figure supplement 8**

Analysis – (A – B) The percentage of flies in each experiment engaging in aggressive behaviors.

All are biological replicates and data are representative of at least two independent trials per experiment. Sample size is displayed per experiment and each experiment consists of approximately 15 flies.

| Figure | Condition | # | Sample Size | Statistical  Test | Comparison | P-value |
| --- | --- | --- | --- | --- | --- | --- |
| 4.8B | EmptySS>Chrimson - | 1 | 7 | Kruskal-Wallis | During: |  |
|  | pC1dSS1>Chrimson -  EmptySS>Chrimson + | 2  3 | 8  8 | (P = 0.0007) | 1 v 2  3 v 4 | >0.9999  0.0030 |
|  | pC1dSS1>Chrimson + | 4 | 9 |  |  |  |
|  |  |  |  |  |  |  |

**Figure 4 – figure supplement 10**

Analysis – (C) Average percentage of flies performing aggressive behaviors over the 30-second period during stimulus delivery. (D) Average percentage of flies performing aggressive behaviors over the 30-second period before 1 Hz, the 60-second periods between subsequent stimuli, and the 30-seconds after 50 Hz stimulation.

All are biological replicates and data are representative of at least two independent trials per experiment. Sample size is displayed per experiment and each experiment consists of approximately 15 flies.

| Figure | Condition | # | Sample Size | Statistical  Test | Comparison | P-value |
| --- | --- | --- | --- | --- | --- | --- |
| 4.10C | Empty>Chrimson 5Hz | 1 | 3 | Mann-Whitney | 1 v 2 | 0.2500 |
|  | pC1dSS1>Chrimson 5Hz  EmptySS>Chrimson 10Hz | 2  3 | 5  3 | Mann-Whitney | 3 v 4 | 0.3929 |
|  | pC1dSS1>Chrimson 10Hz  EmptySS>Chrimson 20Hz  pC1dSS1>Chrimson 20Hz  Empty>Chrimson 30Hz  pC1dSS1>Chrimson 30Hz  Empty>Chrimson 50Hz  pC1dSS1>Chrimson 50Hz | 4  5  6  7  8  9  10 | 5  3  5  3  5  3  5 | Mann-Whitney  Mann-Whitney  Mann-Whitney | 5 v 6  7 v 8  9 v 10 | 0.0357  0.0357  0.0714 |
|  |  |  |  |  |  |  |

| Figure | Condition | # | Sample Size | Statistical  Test | Comparison | P-value |
| --- | --- | --- | --- | --- | --- | --- |
| 4.10D | Empty>Chrimson 10-Hz | 1 | 3 | Mann-Whitney | 1 v 2 | 0.1429 |
|  | pC1dSS1>Chrimson 10-Hz  EmptySS>Chrimson 20-Hz | 2  3 | 5  3 | Mann-Whitney | 3 v 4 | 0.5714 |
|  | pC1dSS1>Chrimson 20-Hz  EmptySS>Chrimson 30-Hz  pC1dSS1>Chrimson 30-Hz  Empty>Chrimson 50-Hz  pC1dSS1>Chrimson 50-Hz | 4  5  6  7  8 | 5  3  5  3  5 | Mann-Whitney  Mann-Whitney | 5 v 6  7 v 8 | 0.3929  0.0357 |
|  |  |  |  |  |  |  |

**Figure 4 – figure supplement 11**

Analysis – (A – C) Total time an individual spent performing aggressive behaviors in a 16 mm arena over the 30 second period prior to or during stimulation. (A' – C') Amount of time during a 30 second 0.1 mW/mm^2^ continuous stimulation period until first aggressive encounter.

All are biological replicates and data are representative of at least two independent trials per experiment.

| Figure | Condition | # | Sample Size | Statistical  Test | Comparison | P-value |
| --- | --- | --- | --- | --- | --- | --- |
| 4.11A | EmptySS>Chrimson | 1 | 22 | Mann-Whitney | 1 v 2 | <0.0001 |
|  | pC1dSS1>Chrimson | 2 | 22 |  |  |  |
|  |  |  |  |  |  |  |

| Figure | Condition | # | Sample Size | Statistical  Test | Comparison | P-value |
| --- | --- | --- | --- | --- | --- | --- |
| 4.11B | EmptySS>Chrimson | 1 | 8 | Mann-Whitney | 1 v 2 | 0.0004 |
|  | pC1dSS1>Chrimson | 2 | 8 |  |  |  |
|  |  |  |  |  |  |  |

| Figure | Condition | # | Sample Size | Statistical  Test | Comparison | P-value |
| --- | --- | --- | --- | --- | --- | --- |
| 4.11C | EmptySS>Chrimson | 1 | 7 | Mann-Whitney | 1 v 2 | 0.0084 |
|  | pC1dSS1>Chrimson | 2 | 8 |  |  |  |
|  |  |  |  |  |  |  |

| Figure | Condition | # | Sample Size | Statistical  Test | Comparison | P-value |
| --- | --- | --- | --- | --- | --- | --- |
| 4.11A' | EmptySS>Chrimson | 1 | 22 | Mann-Whitney | 1 v 2 | <0.0001 |
|  | pC1dSS1>Chrimson | 2 | 22 |  |  |  |
|  |  |  |  |  |  |  |

| Figure | Condition | # | Sample Size | Statistical  Test | Comparison | P-value |
| --- | --- | --- | --- | --- | --- | --- |
| 4.11B' | EmptySS>Chrimson | 1 | 8 | Mann-Whitney | 1 v 2 | 0.0014 |
|  | pC1dSS1>Chrimson | 2 | 8 |  |  |  |
|  |  |  |  |  |  |  |

| Figure | Condition | # | Sample Size | Statistical  Test | Comparison | P-value |
| --- | --- | --- | --- | --- | --- | --- |
| 4.11C' | EmptySS>Chrimson | 1 | 7 | Mann-Whitney | 1 v 2 | 0.0126 |
|  | pC1dSS1>Chrimson | 2 | 8 |  |  |  |
|  |  |  |  |  |  |  |

**Figure 4 – figure supplement 12**

Analysis – Total time an individual spent performing aggressive behaviors over a 30-minute trial.

All are biological replicates and data are representative of at least two independent trials per experiment.

| Figure | Condition | # | Sample Size | Statistical  Test | Comparison | P-value |
| --- | --- | --- | --- | --- | --- | --- |
| 4.12 | pC1dSS1>GFP | 1 | 42 | Kruskal-Wallis | 1 v 2 | 0.1437 |
|  | EmptySS>TNTe  pC1dSS1>TNTe | 2  3 | 28  24 | (P = 0.1156) | 1 v 3  2 v 3 | 0.5411  >0.9999 |
|  |  |  |  |  |  |  |

**Figure 4 – figure supplement 13**

Analysis – (B) Average percentage of flies engaging in aggressive behaviors over the 30-second period prior to or during stimulus delivery.

All are biological replicates and data are representative of at least two independent trials per experiment. Sample size is displayed per experiment and each experiment consists of approximately 15 flies.

| Figure | Condition | # | Sample Size | Statistical  Test | Comparison | P-value |
| --- | --- | --- | --- | --- | --- | --- |
| 4.13B | EmptySS>Chrimson | 1 | 6 | Mann-Whitney | During: |  |
|  | pC1eSS1>Chrimson | 2 | 6 |  | 1 v 2 | 0.0649 |
|  |  |  |  |  |  |  |

**Figure 9B, E**

Analysis – (B) Fraction of time individuals spent performing aggressive behaviors during the stimulus period. (E) Mean length of classified aggressive behavioral events during the stimulus period.

| Figure | Condition | # | Sample Size | Statistical  Test | Comparison | P-value |
| --- | --- | --- | --- | --- | --- | --- |
| 9B | aIPgSS1>Chrimson_L | 1 | 8 | Mann-Whitney | During: |  |
|  | aIPgSS1>Chrimson_D | 2 | 8 |  | 1 v 2 | 0.0019 |
|  |  |  |  |  |  |  |

| Figure | Condition | # | Sample Size | Statistical  Test | Comparison | P-value |
| --- | --- | --- | --- | --- | --- | --- |
| 9E | aIPgSS1>Chrimson_L | 1 | 8 | Mann-Whitney | During: |  |
|  | aIPgSS1>Chrimson_D | 2 | 8 |  | 1 v 2 | 0.0070 |
|  |  |  |  |  |  |  |
